# On the mechanism of carrier recombination in downsized blue micro-LEDs

**DOI:** 10.1038/s41598-021-02293-0

**Published:** 2021-11-23

**Authors:** Po-Wei Chen, Po-Wen Hsiao, Hsuan-Jen Chen, Bo-Sheng Lee, Kai-Ping Chang, Chao-Chun Yen, Ray-Hua Horng, Dong-Sing Wuu

**Affiliations:** 1 Department of Materials Science and Engineering & Innovation and Development Center of Sustainable Agriculture, National Chung Hsing University, Taichung, 40227 Taiwan; 2 Epileds Technologies, Incorporated, Tainan, 74148 Taiwan; 3 Institute of Electronics, National Yang Ming Chiao Tung University, Hsinchu, 30010 Taiwan; 4 Department of Applied Materials and Optoelectronic Engineering, National Chi Nan University, Nantou, 54561 Taiwan

**Keywords:** Engineering, Materials science, Nanoscience and technology, Optics and photonics, Physics

## Abstract

The mechanism of carrier recombination in downsized μ-LED chips from 100 × 100 to 10 × 10 μm^2^ on emission performance was systemically investigated. All photolithography processes for defining the μ-LED pattern were achieved by using a laser direct writing technique. This maskless technology achieved the glass-mask-free process, which not only can improve the exposure accuracy but also save the development time. The multi-functional SiO_2_ film as a passivation layer successfully reduced the leakage current density of μ-LED chips compared with the μ-LED chips without passivation layer. As decreasing the chip size to 10 × 10 μm^2^, the smallest chip size exhibited the highest ideality factor, which indicated the main carrier recombination at the high-defect-density zone in μ-LED chip leading to the decreased emission performance. The blue-shift phenomenon in the electroluminescence spectrum with decreasing the μ-LED chip size was due to the carrier screening effect and the band filling effect. The 10 × 10 μm^2^ μ-LED chip exhibited high EQE values in the high current density region with a less efficiency droop, and the max-EQE value was 18.8%. The luminance of 96 × 48 μ-LED array with the chip size of 20 × 20 μm^2^ exhibited a high value of 516 nits at the voltage of 3 V.

## Introduction

Self-emissive light-emitting diode (LED) devices can be divided into two major technologies, which are inorganic-based and organic-based technologies. Both technologies possessed fast response time and color saturation properties^1–3^. For display applications, the organic LED display has been already widely applied on smartphones and televisions. However, the organic LED display was hard to operate in high-temperature conditions and high injection current for a long-time, leading to limit the applications in high brightness and humid tropical condition^4,5^. The inorganic LEDs not only can operate in high injection current for high brightness applications but have superior stability in humid tropical conditions compared with organic LEDs^6–8^. For achieving a high-resolution display, the number of chips in the unit area should be increased for increasing the pixel density. Therefore, the downsizing in the chip size was a necessary and efficient way to increase the pixel density^9,10^. As decreasing in the LED size to the micron scale, it was called the micro-LED (μ-LED). However, the performance of μ-LED chip decreased with decreasing the chip size, which was due to the increased non-radiative recombination at sidewall defects from the dry etching induced damage. Since the MESA and isolation process of μ-LED chip was achieved by using the inductively coupled plasma reactive ion etch system (ICP-RIE), the ion bombardment during the high plasma power etching process created the surface defects on the sidewalls of μ-LED chip^8,11,12^. Therefore, for improving and analyzing the light extraction performance of μ-LED chips and arrays, this study was divided into three parts as the reducing leakage current by inserting a passivation layer, the analysis in the size-dependent effect on various μ-LED chips, and the analysis in the μ-LED array with a 20 × 20 μm^2^ chip size. Firstly, the dielectric materials such as the SiO_2_ or the Al_2_O_3_ having the wide bandgap property and the low refraction index property have high potential as a passivation layer^13,14^. The SiO_2_ film was employed as a passivation layer in this study, which was deposited by using plasma-enhanced chemical vapor deposition (PECVD). The SiO_2_ film could be a multi-functional layer in μ-LED including the isolation, planarization, and passivation functions. Secondly, the μ-LED chip size decreased from 100 × 100 to 10 × 10 μm^2^ for improving the display resolution. Furthermore, the downsizing effect of μ-LED chip on the light emission property and mechanism was investigated. Thirdly, the 96 × 48 μ-LED array with a chip size of 20 × 20 μm^2^ was analyzed on the 3D beam profile and luminance.

In this study, the photolithography processes for defining the μ-LED pattern were achieved by using a laser direct writing technique. This system not only can reduce the use of glass masks, but also can improve the exposure accuracy and save the development time. The effect of the passivation layer on the leakage current density was studied. The effect of downsizing in the chip size on the output power density, external quantum efficiency (EQE), and electroluminescence was systemically investigated. The mechanism of carrier recombination in downsized μ-LED was discussed. The performance of μ-LED array with a 20 × 20 μm^2^ chip size was also studied.

## Results and discussion

### Reducing leakage current by a SiO_2_ passivation layer

**Figure 1:**
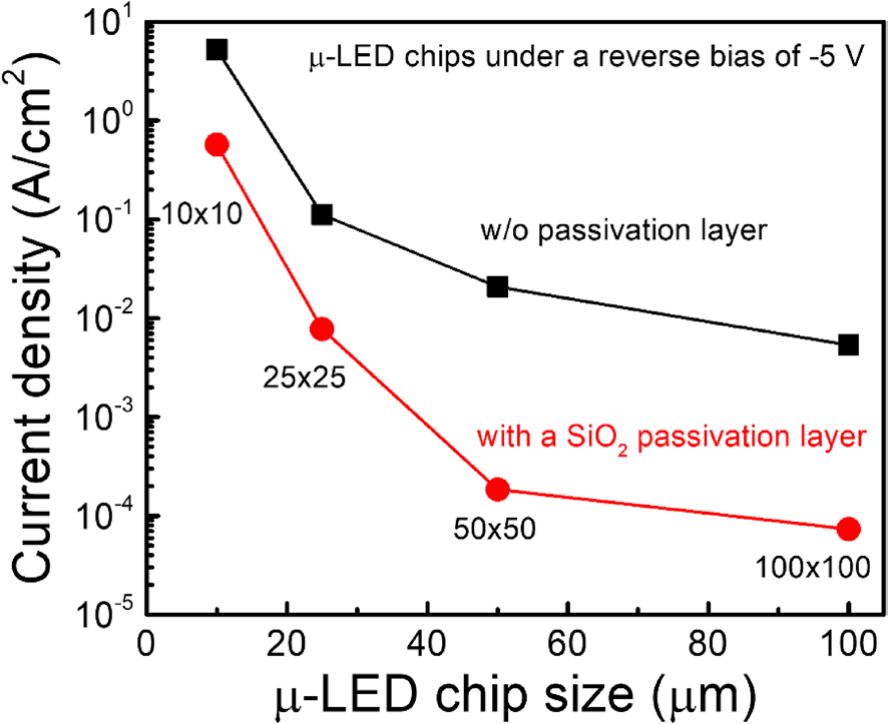
The current density of μ-LED chips with and without SiO_2_ passivation layer as a function of chip size. The passivation layer was deposited on the μ-LED chips after the MESA process.

For reducing the leakage current from the sidewall defects of μ-LED chips, the SiO_2_ passivation layer was deposited on the μ-LED chips after the MESA step by using PECVD. Although the passivating step in the fabrication process (as shown in Fig. 8d) of μ-LED chips was performed after the isolation step, the SiO_2_ passivation layer was deposited after the MESA step for analyzing the effect of passivated ability via a single dry etching process. Figure 1 shows the current density of μ-LED chips with and without SiO_2_ passivation layer as a function of chip size. After depositing the SiO_2_ passivation layer on the μ-LED chip, the leakage current density of chips decreased dramatically, which exhibited the SiO_2_ passivation layer providing a good passivation ability. This was because the SiO_2_ film can passivate the surface dangling bonds on the μ-LED chips, leading to the reduced leakage current^15^. As decreasing the chip size from 100 × 100 to 10 × 10 μm^2^, the leakage current of both chip series with and without passivation layer increased. This was because the sidewall area decreased with decreasing the chip size, resulting in the increased sidewall defect density. This leads to increased leakage current density. As decreasing the chip size, the leakage current density of 100 × 100 μm^2^ chip after covering a passivation layer decreased from 5.35 × 10^3^ to 7.36 × 10^5^ A/cm^2^, which had a hundred times difference. Furthermore, the leakage current density of chip size of 10 × 10 μm^2^ after passivation reduced from 5.23 to 0.57 A/cm^2^, which showed a ten times difference. The SiO_2_ passivation ability in the chip size of 10 × 10 μm^2^ was inferior to that of the chip size of 100 × 100 μm^2^. This was probably because the shape of the smallest chip size become sharper, leading to the deteriorated coverage of passivation layer. Besides, the SiO_2_ insulating layer was acted as a multi-functional layer including the isolation, planarization, and passivation functions. The isolation function of SiO_2_ layer isolated the anode and cathode metal electrodes, which was due to the insulated property of SiO_2_ layer. Since the thickness of the SiO_2_ layer was over 500 nm, this can reduce the step-height difference between the anode and cathode leading to smooth the metal contact. Therefore, the planarization function can be achieved.

### Size-dependent effect on μ-LED chips

**Figure 2:**
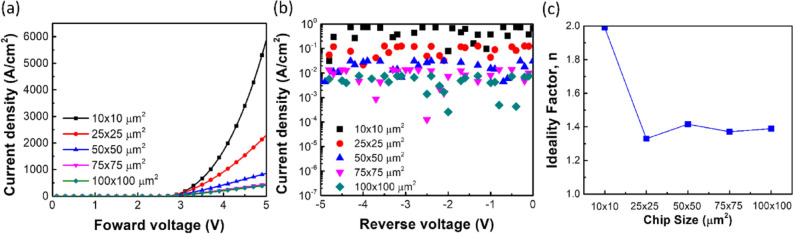
(**a**) The current density of μ-LED chip with various chip size as a function of forward voltage. (**b**) The current density of μ-LED chip with various chip size as a function of reverse voltage. (**c**) The ideality factor n of μ-LED chip as a function of chip size.

**Figure 3:**
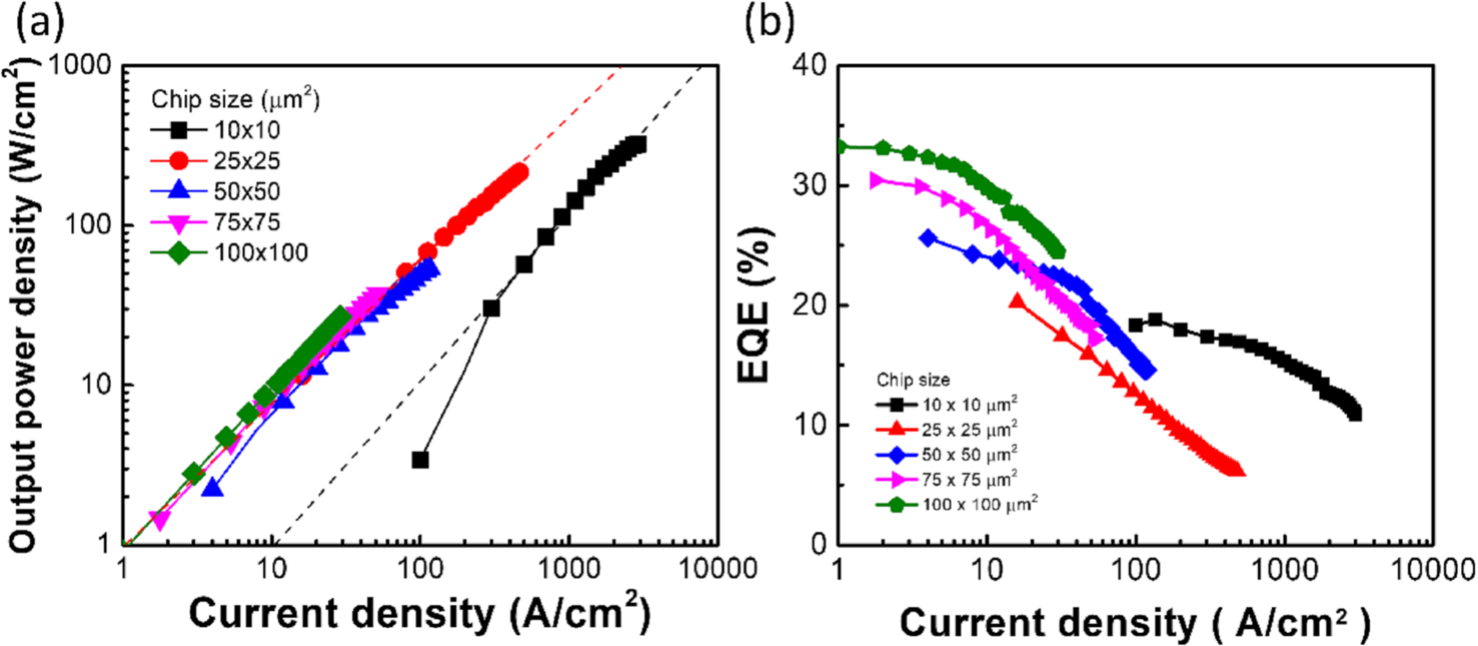
(**a**) The output power density as a function of current density with various μ-LED chip size. Dash lines are drawn to guide the eyes. (**b**) The EQE as a function of current density with various μ-LED chip size.

Figure 2a shows the current density with various chip sizes as a function of forward voltage. As decreasing the chip size from 100 × 100 to 10 × 10 μm^2^, the current density increased from 36.5 to 114.5 A/cm^2^. The reduced chip size led to the increased current density inside the μ-LED chip. In addition, the current density under the reverse bias voltage from 0 to − 5 V shows rather stable, as shown in Fig. 2b. As decreasing the chip size from 100 × 100 to 10 × 10 μm^2^, the current density under the bias of − 5 V increased from − 0.329 to − 0.003 A/cm^2^, which indicated the released undesired current from defect states^16^. By the way, the reversed current density of 50 × 50 μm^2^ chip size in Fig. 2b was higher than the reversed current density in Fig. 1, which was because the passivation layer was deposited after the isolation step. The chip after the isolation step means the chip pass through two-times dry etching process. Therefore, the sidewall defect density after the isolation step should be higher than the chip after the MESA step. These defect states in the energy diagram are normally located nearly by the conduction band and valence band so that the carrier hops through these states under the reverse bias^17^. Furthermore, these defect states were mostly created during the dry etching process because the high-energy ions in the plasma bombarded the epitaxial area without a photoresist mask. The high-energy atoms collided with the epitaxial surface, leading to creating the dangling bond with an unpaired electron. Therefore, the injected current under the forward bias was trapped by defect states, resulting in the reduced conversion efficiency (as shown later in Fig. 3b). In contrast, under the reverse bias, the trapped electrons or holes were ejected from defect states forming the leakage current.

The recombination mechanisms in a p–n junction include two different models such as the band-to-band radiative recombination and the Shockley–Read–Hall (SRH) nonradiative recombination^18^. The band-to-band radiative recombination is the main emission mechanism in the LED by an injected current, which can produce a photon via the spontaneous emission. Besides, the SRH nonradiative recombination is normally induced by the defect state, which led to the increased leakage current and the reduced conversion efficiency^19^. Both recombination mechanisms can be further analyzed by an ideality factor n in Shockley diode equation, as shown in Eq. (1):

1 $$I={I}_{s}\left({e}^\frac{qV}{nkT}-1\right)$$

where *I* is the diode current, *I*_*s*_ is the saturation current at the reverse voltage, *V* is the voltage over the diode, *q* is the elementary charge, *k* is the Boltzmann constant, and *T* is the temperature in kelvins. Figure 2c shows the ideality factor n of μ-LED chip as a function of chip size. As decreasing the chip size from 100 × 100 to 25 × 25 μm^2^, the ideality factors exhibit the similar value around 1.3 to 1.4. The increased ideality factor from 1.33 to 1.99 with decreasing the chip size from 25 × 25 to 10 × 10 μm^2^. Furthermore, the ideality factor of 1.0 is indicated that the band-to-band radiative recombination dominated the recombination mechanism in the μ-LED chip. In contrast, the ideality factor of 2.0 is indicated that the SRH nonradiative recombination dominated via defect states^20,21^. Therefore, the smallest chip size exhibited the highest ideality factor, which indicated the main carrier recombination at the high-defect-density zone in the μ-LED chip. The high-defect-density zone in the smallest chip size of μ-LED was located at the chip’s sidewall, which has a short distance from the sidewall to the main carrier recombination center. Thus, the light conversion efficiency decreased. However, the large chip size μ-LED exhibited less influence on conversion efficiency because the main carrier recombination center is far from the sidewalls. As decreasing the chip size, the chip sidewalls are approached the center of μ-LED chip, leading to the shifted recombination zone to the μ-LED sidewalls.

Figure 3a shows the output power density as a function of current density with various μ-LED chip sizes. As decreasing the μ-LED chip size from 100 × 100 to 25 × 25 μm^2^, the output power density increased with increasing the current density. The output power density of the smallest μ-LED chip revealed a lower value than those of larger chips under the same current density. Figure 3b shows the external quantum efficiency (EQE) as a function of current density with various μ-LED chip sizes. The max-EQE decreased from 33.3 to 18.8% with decreasing the μ-LED chip size from 100 × 100 to 10 × 10 μm^2^. This decrease in EQE was attributed to the increased defect-related non-radiative recombination^22–24^. Since the injected carrier density on a unit area of μ-LED chip increased with reducing the chip size, the output power per unit area should be improved. However, as decreasing chip size from 100 × 100 to 25 × 25 μm^2^, the output power per unit area under the same injected-carrier-density possessed a similar value, which was due to the decreased EQE. Furthermore, the EQE can be further analyzed by an ABC model^25–27^.

2 $${\eta }_{EQE}=\frac{{\eta }_{extract}\times B{n}^{2}}{\left(An+{Bn}^{2}+{Cn}^{3}\right)}$$

where η_extract_ is the light extraction efficiency, n is the carrier concentration, A indicates the SRH nonradiative recombination coefficient, B indicates the bimolecular radiative coefficient, and C indicates the Auger non-radiative recombination coefficient. The B coefficient is mainly related to the max-EQE because of the proportion of n^2^. The decreased max-EQE was due to the reduced spontaneous emission. Furthermore, the SRH nonradiative recombination also influenced the max-EQE value. The point of max-EQE is located when the dη_EQE_/dn = 0. Thus, the carrier concentration n can be obtained as n = (A/C)^1/2^ from Eq. (2). The decreased max-EQE with shifting toward high current density indicated the increased A coefficient or the decreased C coefficient. For increase in the A coefficient, this is presented the increased SRH nonradiative recombination. The main influence of SRH recombination from sidewall defects became obvious with reducing μ-LED chip size. Since the area of μ-LED chip became smaller, the injected current could spread across the whole μ-LED chip including the sidewall area^23^. Therefore, the max-EQE of a smaller chip size was significantly influenced by sidewall defects. Besides, the decreased C coefficient with reducing the chip size was due to the reduced Auger recombination, which also induced the reduced efficiency droop in the high current density region^12,25,28^. The Auger recombination indicated that the carriers are generated into higher energy levels within the same band. Thus, the widened bandgap of multi-quantum wells can suppress the Auger recombination, which was due to reducing the generated carrier toward higher energy levels^25^. However, the material of μ-LED chips in this study was consistent. Therefore, the possible reason was the band filling effect occurring with decreasing the μ-LED chip size, leading to the accumulated carriers at the conduction band and the valance band as increasing the current density. The electron–hole pair may recombine from a higher energy level above the conduction band to a lower energy level below the valance band, which is similar to the widened bandgap. At the chip size of 50 × 50 μm^2^, the curve was under the transition period leading to the curve in the high current density region still having an efficiency droop effect. Therefore, the EQE curve became more linear after the chip size of 50 × 50 μm^2^. As a result, the smallest μ-LED chip exhibited a less efficiency droop effect, but the max-EQE value was low.

**Figure 4:**
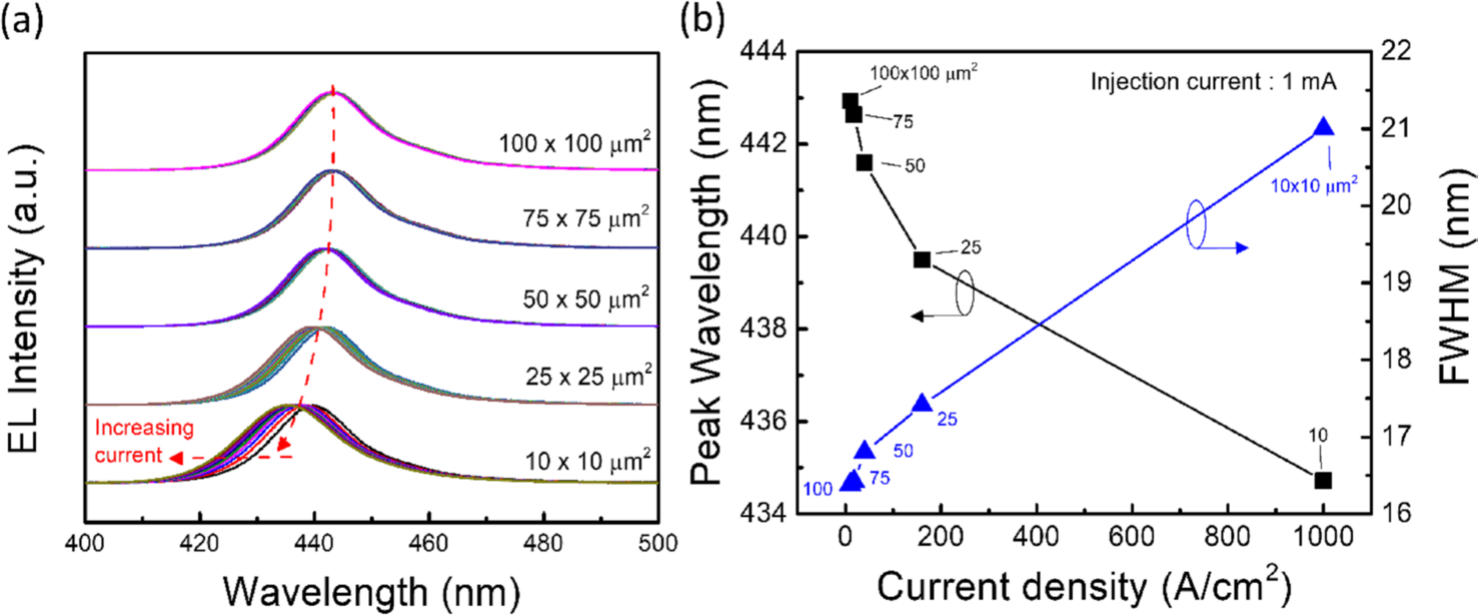
(**a**) The electroluminescence (EL) spectra after normalization as a function of wavelength with various μ-LED chip size. The injected current increased from 0.1 to 1 mA. (**b**) The peak wavelength and FWHM of electroluminescence spectra as a function of current density with various μ-LED chip size. The injection current was 1 mA. The square symbol indicates the peak wavelength and the triangle symbol indicates the FWHM.

**Figure 5:**
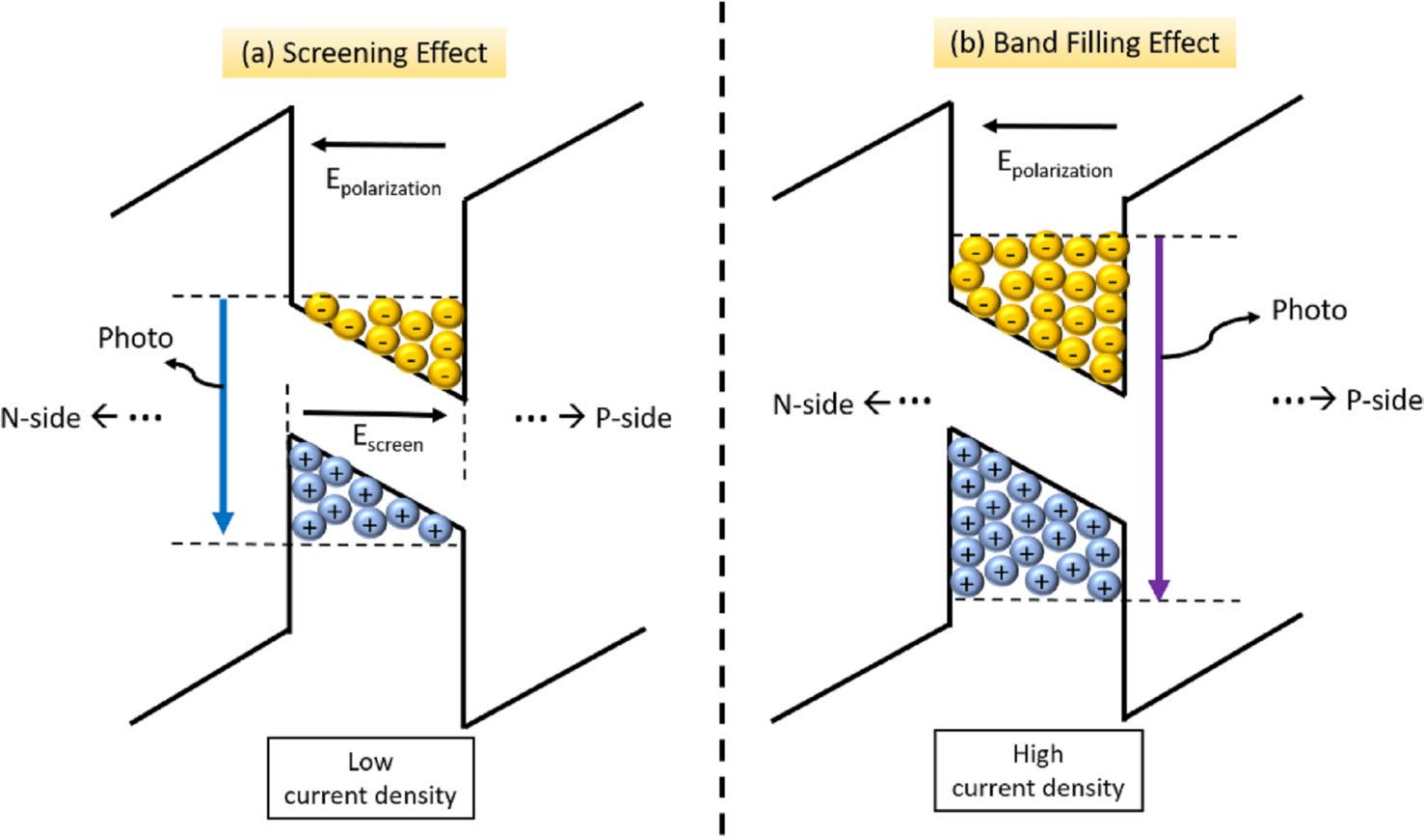
The schematic of band diagram for (**a**) screening effect and (**b**) band filling effect in the μ-LED chip.

Figure 4a shows the electroluminescence spectrum after normalization as a function of wavelength with various μ-LED chip sizes. As decreasing the μ-LED chip size from 100 × 100 to 50 × 50 μm^2^, the peak-wavelength of emitted light was located at 443 ± 1 nm with increasing the injected current from 0.1 to 1 mA. Furthermore, the μ-LED chip size of 25 × 25 and 10 × 10 μm^2^ exhibited a blue-shift phenomenon from 442 to 439 nm and from 439 to 434 nm with increasing the injected current, respectively. This blue-shift phenomenon was attributed to two possible reasons such as the carrier screening effect and the band filling effect^29^. Furthermore, both the carrier screening effect and the band filling effect in μ-LED can be further analyzed by the peak wavelength and full width at half maximum (FWHM) of electroluminescence spectra under the same injection current of 1 mA, which was plotted as a function of current density with various μ-LED chip size, as shown in Fig. 4b. As decreasing the chip size from 100 × 100 to 50 × 50 μm^2^, the peak wavelength slightly blue-shifted about 1.35 nm with corresponding current density from 10 to 40 A/cm^2^. Meanwhile, the FWHM widened only 0.5 nm. These phenomena may cause by the carrier screening effect, which was because the injected carriers accumulated at the band edge in quantum well inducing the carrier screening electrical field compensating the polarization electrical field^30^. Thus, the transition of electron–hole pair from higher energy level resulted in the slight blue-shift of the luminescence. The schematic of the band diagram for the carrier screening effect is shown in Fig. 5. With decreasing the chip size from 50 × 50 to 10 × 10 μm^2^, the peak wavelength significantly blue-shifted about 6 nm, and the FWHM widened about 4.2 nm with corresponding current density from 40 to 1000 A/cm^2^. This was because the dramatic increased current density filled the energy band from the bottom edge of multi-quantum wells leading to the transition of electron–hole pair from a higher energy level above the occupied states in the conduction band to a lower energy level below the occupied states in the valance band, as shown in Fig. 5. As a result, this band-filling effect led to the obvious blue shift of the luminescence^30,31^. Furthermore, the multi-quantum wells near the p-side benefited more holes accumulation than near the n-side^29^. Therefore, the band filling effect may gradually degrade to the n-side, which led to the broad FWHM of luminescence peak.

### μ-LED array with 20 × 20 μm^2^ chip size

**Figure 6:**
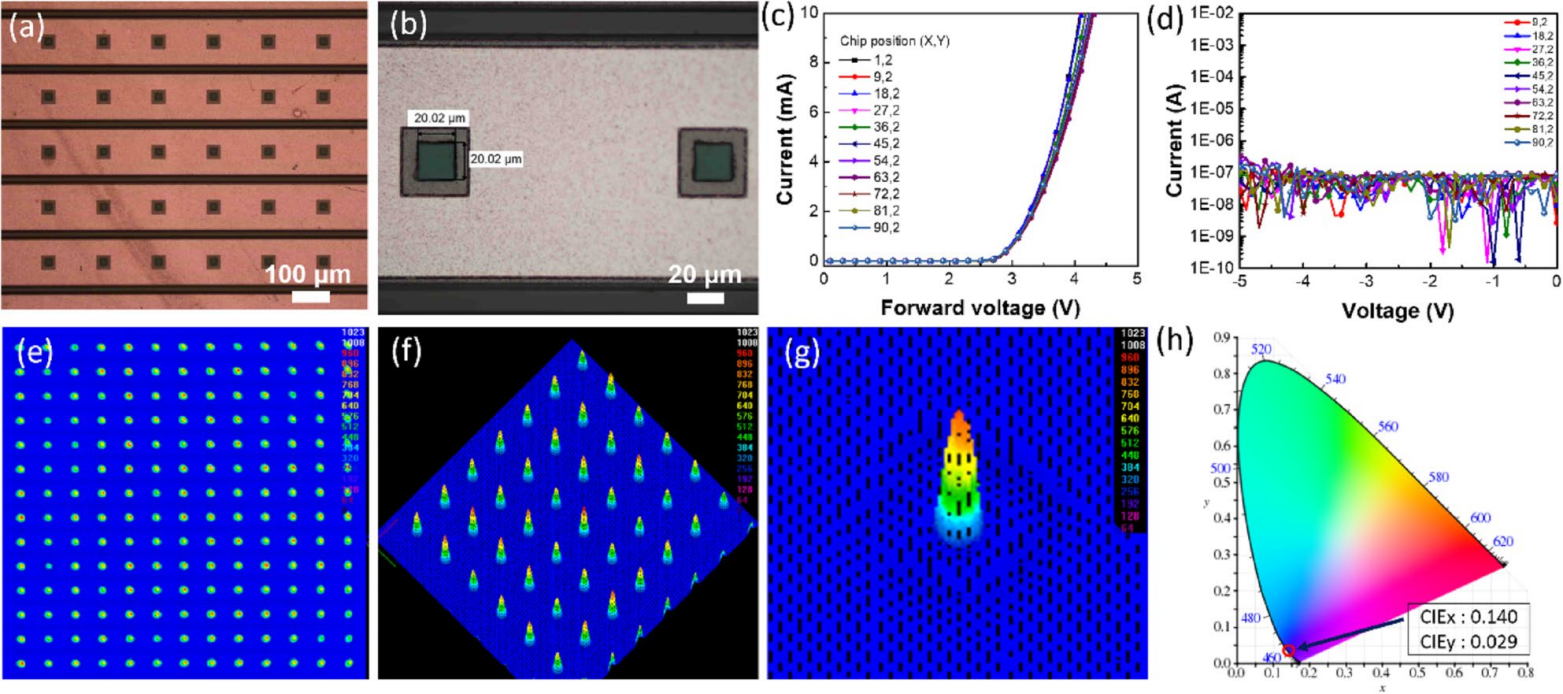
The 96 × 48 μ-LED array was analyzed by (**a**) optical micrographs of array and (**b**) chips with 20 × 20 μm^2^, I–V curve of chips in a single row of array under (**c**) the forward voltage and (**d**) the reverse voltage, (**e**) the 2D and (**f**) 3D beam profiles of a part of array, (**g**) 3D beam profiles of a chip, and (**f**) the CIE1931 color space for evaluating the emission color (red circle).

Since the μ-LED chip with a chip size of 10 × 10 μm^2^ exhibited the lowest max-EQE at the low injection current density and the most blue-shifted emission wavelength at the high injection current density, the 25 × 25 μm^2^ μ-LED chip size could be the most potential for the minimized chip size with the acceptable emission performance. Therefore, we designed a 96 × 48 μ-LED array with the chip size of 20 × 20 μm^2^ for further reaching the high resolution of 171 PPI. Figure 6a,b show the optical micrographs of a part of 96 × 48 μ-LED array and μ-LED chips with the size of 20 × 20 μm^2^, respectively. Figure 6c shows the I–V curve of chips in a single row of 96 × 48 μ-LED array. The forward voltage under the current of 1 mA was 3.1 V. Under the reverse voltage of − 5 V, the leakage current was 400 nA, as shown in Fig. 6d. Furthermore, the I–V curve with different sites in a column exhibited low deviation, which indicated the high uniformity of μ-LED array. Figure 6e,f show the 2D and 3D beam profiles of a part of 96 × 48 μ-LED array under the turn-on state, respectively. The part of the μ-LED array exhibited a high luminance uniformity. As shown in Fig. 6g, the 3D beam profiles of a signal chip with a size of 20 × 20 μm^2^ exhibited the highest luminance intensity at the center of chip, which indicated the main carrier recombination at the center. Furthermore, Fig. 6h shows the emission color in the CIE1931 chromaticity diagram. The coordinate of μ-LED array under the voltage of 3 V was located at the CIE_x_ of 0.140 and CIE_y_ of 0.029 indicating the blue color. As a result, the luminance of μ-LED array exhibited a high value of 516 nits at the voltage of 3 V.

## Conclusion

In this study, all photolithography processes for defining the μ-LED pattern were achieved by using a laser direct writing technique, which can save the use of glass masks and the development time. The impact of downsized μ-LED chips from 100 × 100 to 10 × 10 μm^2^ on the emission performance and the spectral shift was discussed. The reduced leakage current density was achieved by employing a multi-functional SiO_2_ passivation layer, which decreased dramatically from 10^−1^ to 10^−3^ A/cm^2^ level. The chip size of 10 × 10 μm^2^ exhibited the highest ideality factor of 1.99, which indicated the main carrier recombination at the high-defect-density zone in μ-LED chip leading to the decreased emission performance. As increasing the injected current, the μ-LED chip size of 25 × 25 and 10 × 10 μm^2^ exhibited blue-shift phenomenon, which were attributed to the carrier screening effect and the band filling effect. The μ-LED chip size of 10 × 10 μm^2^ exhibited high EQE values in the high current density region with a less efficiency droop, which was due to the decreased Auger recombination. The max-EQE decreased from 33.3 to 18.8% with decreasing the μ-LED chip size from 100 × 100 to 10 × 10 μm^2^. For μ-LED array, the 96 × 48 μ-LED array with the chip size of 20 × 20 μm^2^ was achieved with a good luminance uniformity. The forward voltage under the current of 1 mA was 3.1 V and the luminance under the voltage of 3 V was 516 nits.

## Methods

The μ-LED chip and array were fabricated from the blue LED epilayer with a peak wavelength of 450 ± 5 nm. The blue LED epilayers were grown on sapphire substrate by metal–organic chemical vapor deposition (MOCVD). The epilayers from the substrate to surface is the undoped GaN buffer layer (1.55 μm), n-type GaN/AlGaN current spreading layer (1.42 μm), InGaN/GaN stacked multiple quantum wells (MQW, 0.6 μm), and p-type AlGaN/GaN/InGaN electron blocking layer (0.4 μm). The tin-doped indium oxide (ITO, 0.28 μm) was deposited on the p-type GaN for ohmic contacts by e-beam evaporation.

**Figure 7:**
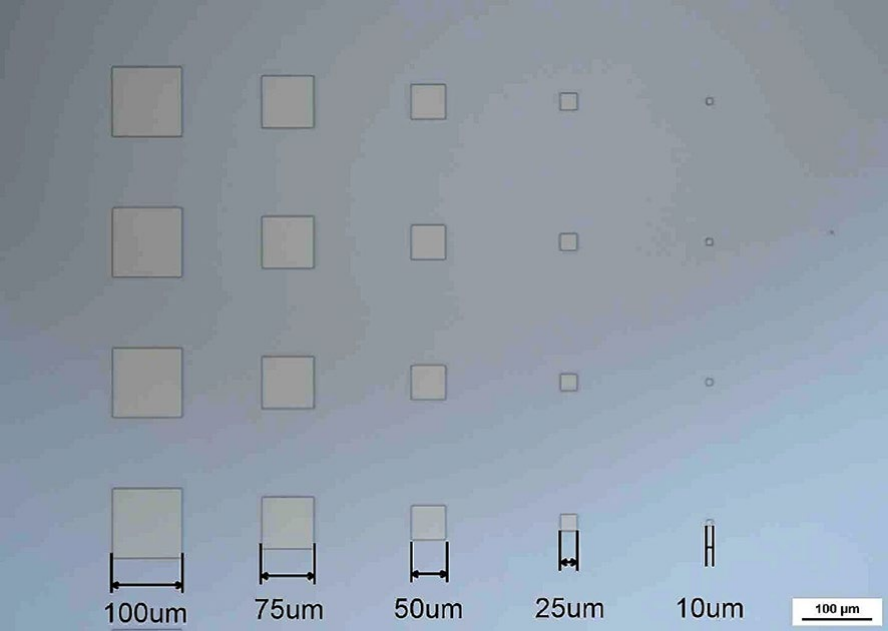
The optical micrograph of μ-LED chips with various chip size from 100 × 100 to 10 × 10 μm^2^ designed on a 4-inch epitaxial wafer.

**Figure 8:**
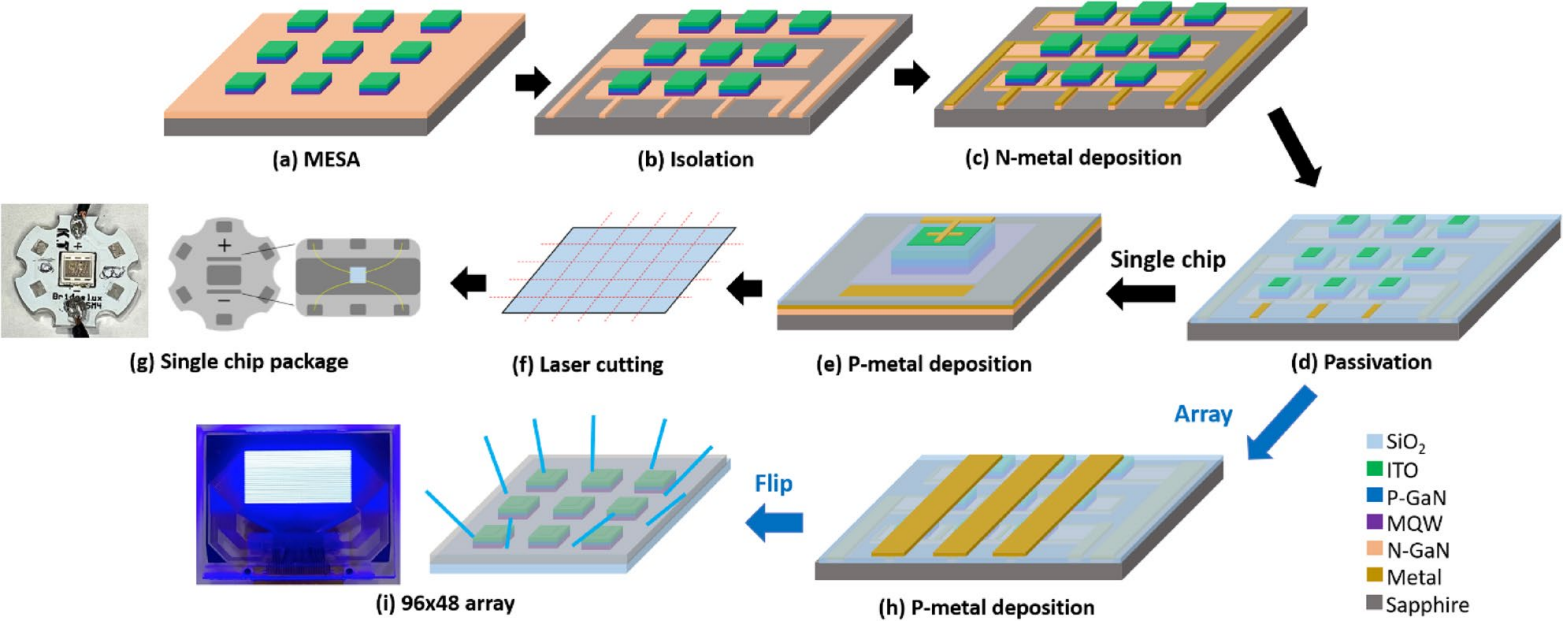
The fabrication flow of μ-LED chip and array included the mutual part of (**a**) MESA, (**b**) isolation, (**c**) N-metal deposition, and (**d**) passivation. Then, the fabrication flow was separated into two processes for fabricating single chip and array. The processes of (**e**) P-metal deposition, (**f**) laser cutting, and (**g**) package were for fabricating a single chip. The processes of (**h**) P-metal deposition was for fabricating a (**i**) 96 × 48 array.

The μ-LED chips with various chip sizes were designed on a 4-in. epitaxial wafer, which can suppress the deviation between wafer by wafer, as shown in Fig. 7. The manufacturing steps of the μ-LED chip and array are shown in Fig. 8. The μ-LED chips and arrays were fabricated by using photolithography processes, etching processes, and metal deposition processes. The photolithography processes defined the pattern parts of each step for further etching process or deposition process. In this study, we used a laser direct writing system (Heidelberg Instruments, MLA-150) for the exposure in photolithography processes. This equipment using the laser light directly exposes the photoresist to transfer the pattern, which increases the precision of the lithography process and achieves the maskless process. The epilayers for MESA (Fig. 8a) and isolation (Fig. 8b) processes were etched by using the ICP-RIE system with reactive gases of Cl_2_, BCl_3_, Ar, and CHF_3_. Then, the n-interconnect metal (Fig. 8c) of Cr/Pt/Au (5/30/600 nm) on the n-GaN layer was deposited by using an e-beam evaporation. Afterward, the SiO_2_ film was employed as a passivation layer (Fig. 8d), which was deposited by PECVD for planarization, electrode isolation, and passivation sidewall of chips. The thickness of passivation layer was 525 nm. The p-metal (Fig. 8e,h) of Cr/Pt/Au (3000 nm) was deposited by using the e-beam evaporator. The single-chip was split by laser cutting (Fig. 8f) before packaging on a printed circuit board (Fig. 8g). The μ-LED array (Fig. 8i) with the chip size of 20 × 20 μm^2^ is 96 × 48 chips with a diagonal resolution of 171 dots per inch. The current–voltage (I–V) curve, output power, and EQE were measured by the IV measurement system with an integrating sphere.

The IV curve, forward voltage (V_f_), and EQE were measured by the IV measurement system with an integrating sphere. The electroluminescence spectra were measured by an electroluminescence system with the injected current from 0.1 to 1 mA. The 3D image of μ-LED array was measured by a beam profiler system with a full-frame Progressive Scan CCD camera (COHU 6612-1000). The luminance of μ-LED array was measured by a chroma meter (Minolta CS-100) in the dark box.
